# Four New Patient-Reported Outcome Measures Examining Health-Seeking Behavior in Persons With Type 2 Diabetes Mellitus (REDD-CAT): Instrument Development Study

**DOI:** 10.2196/63434

**Published:** 2024-11-22

**Authors:** Suzanne E Mitchell, Michael A Kallen, Jonathan P Troost, Barbara A De La Cruz, Alexa Bragg, Jessica Martin-Howard, Ioana Moldovan, Jennifer A Miner, Brian W Jack, Noelle E Carlozzi

**Affiliations:** 1 Department of Family Medicine School of Medicine Boston University Boston, MA United States; 2 Department of Family Medicine Boston Medical Center Boston, MA United States; 3 Department of Family Medicine and Community Health Chan Medical School University of Massachusetts Worcester, MA United States; 4 Department of Medical Social Sciences Feinberg School of Medicine Northwestern University Chicago, IL United States; 5 Michigan Institute for Clinical and Health Research University of Michigan Ann Arbor, MI United States; 6 Department of Physical Medicine and Rehabilitation University of Michigan Ann Arbor, MI United States

**Keywords:** diabetes mellitus, social determinants of health, patient-reported outcome measures, outcomes assessment, health care, patient reported, health-seeking behavior, type 2 diabetes, hospitalization, diabetes computer adaptive test, primary care, socioeconomic, assessments

## Abstract

**Background:**

The management of type 2 diabetes mellitus (T2DM) includes mastery of complex care activities, self-management skills, and routine health care encounters to optimize glucose control and achieve good health. Given the lifelong course of T2DM, patients are faced with navigating complex medical and disease-specific information. This health-seeking behavior is a driver of health disparities and is associated with hospitalization and readmission. Given that health-seeking behavior is a potentially intervenable social determinant of health, a better understanding of how people navigate these complex systems is warranted.

**Objective:**

To address this need, we aimed to develop new patient-reported outcome (PRO) measures that evaluate health-seeking behavior in persons with T2DM. These new PROs were designed to be included in the Re-Engineered Discharge for Diabetes-Computer Adaptive Test (REDD-CAT) measurement system, which includes several other PROs that capture the importance of social determinants of health.

**Methods:**

Overall, 225 participants with T2DM completed 56 self-report items that examined health-seeking behaviors. Classical Test Theory and Item Response Theory were used for measurement development. Exploratory factor analysis (EFA; criterion ratio of eigenvalue 1 to eigenvalue 2 being >4; variance for eigenvalue 1 ≥40%) and confirmatory factor analysis (CFA; criterion 1-factor CFA loading <.50; 1-factor CFA residual correlation >.20; comparative fit index ≥0.90; Tucker-Lewis index ≥0.90; root mean square error of approximation <0.15) were used to determine unidimensional sets of items. Items with sparse responses, low-adjusted total score correlations, nonmonotonicity, low factor loading, and high residual correlations of high error modification indices were candidates for exclusion. A constrained graded response model was used to examine item misfit, and differential item functioning was examined to identify item bias. Cronbach α was used to examine internal consistency reliability for the new PROs (criterion ≥0.70), and floor and ceiling effects were examined (criterion ≤20%).

**Results:**

Four unidimensional sets of items were supported by EFA (all EFA eigenvalue ratios >4; variance for eigenvalue 1=41.4%-67.3%) and CFA (fit statistics all exceeded criterion values). This included (1) “Health-Seeking Behavior: PCP-Specific” (6 items); (2) “Health-Seeking Behavior: General Beliefs” (13 items); (3) “Health-Seeking Behavior: Family or Friends-Specific” (5 items); and (4) “Health-Seeking Behavior: Internet-Specific” (4 items). All items were devoid of differential item functioning for age, sex, education, or socioeconomic status factors. “Health-Seeking Behavior: General Beliefs” was developed to include both a computer adaptive test and a 6-item short form version; all other PROs were developed as static short forms. The psychometric reliability of these new PROs was supported; internal consistency ranged from acceptable to excellent (Cronbach α=.78-.91), and measures were free of significant floor or ceiling effects (floor effects range: 0%-8.9%; ceiling effects range: 0%-8.4%).

**Conclusions:**

The new REDD-CAT Health-Seeking Behavior PROs provide reliable assessments of health-seeking behaviors among those with T2DM.

## Introduction

Over 37 million Americans live with diagnosed diabetes, accounting for 7.8 million hospitalizations and over US $327 billion in annual health care costs [1]. The lifelong course of type 2 diabetes mellitus (T2DM) demands mastery of complex care activities, self-management skills, and routine health care encounters to optimize glucose control and achieve good health. Through evidence-based diabetes self-management education programs, patients gain the essential knowledge, skills, and support to practice diabetes self-care and navigate the health care system as informed and engaged participants in their care [2]. Simultaneously, increased access to online resources and shifts toward shared decision-making models in medicine have promoted a culture where patients can more readily seek and acquire health services, information, and knowledge for themselves [3,4].

Health seeking is broadly characterized as “any activity undertaken by individuals who have a health problem or to be ill for the purpose of finding an appropriate remedy” [5]. Health-seeking behaviors are further contextualized by culture, language, and socioeconomic factors, all of which underscore individual decision-making and behavior change processes in response to illness [5,6]. Comprised of both information-gathering and use of services, health-seeking behaviors have been studied in the context of coping among patients with cancer and other serious illnesses [7], and to a lesser extent, as a marker of patient activation [8,9]. A systematic review of literature on health-seeking behavior concluded that routine assessments of patients’ health-seeking behaviors and attitudes are warranted as a marker of patient activation and healthy coping with illness [4]. The credibility of patients’ information sources and their intent to act on such information should also be considered in the context of their social environment [4,6].

Researchers have identified individual sociodemographic differences in health-seeking behaviors, with higher levels of health literacy and younger age linked to active health-seeking among White adults [7,9,10]. These empiric findings implicate health-seeking behavior as a possible driver of health disparities for those living with diabetes and a possible marker forecasting poor coping with illness situations leading to increased risk for hospitalization and readmission. By nature of their lifelong disease, patients with T2DM rely on disparate sources for medical and disease-specific information, ranging from interpersonal connections, such as friends and family, to health care professionals, as well as more ubiquitous sources, including the internet, television, and social media [11]. In doing so, patients seek to weave a web of information that is relevant and personalized to their needs and experiences, thus influencing their immediate or delayed interactions with health systems [8]. However, few studies on health service attainment and seeking behavior have focused specifically on diabetes care.

The role and patterns of health-seeking behavior as a potentially intervenable social determinant of health remain underexplored in research and medical literature. Understanding the circumstances and patient- and system-level factors that drive such behaviors have clinical implications, both positive (eg, adherence) and negative (eg, nonadherence) [4,6]. In the context of diabetes, health-seeking behavior is a component of diabetes self-management skills. The degree to which an individual masters disease self-management strongly influences the likelihood of serious complications, hospitalizations, and premature death, and reflects the individual level of coping with this serious chronic disease.[2] Thus, there is a valid need to better understand the factors that influence health-seeking behavior and its role as a social determinant of health.

The purpose of these analyses was to develop new patient-reported outcomes (PROs) according to established measurement development standards [12] that assess health-seeking behavior in persons with T2DM. These new measures of health-seeking behavior will be included in part of a larger, more comprehensive measurement system designed to capture important social determinants of health that are related to readmission risk in people with T2D. This measurement system, the Re-Engineered Discharge for Diabetes-Computer Adaptive Test (REDD-CAT), includes PROs that capture personal (health literacy, mood, pain management, stigma, illness burdensomeness, caregiver needs, substance abuse, finances, food, and transportation), social (social support, isolation, and provider connection), and community factors (health care access, medication access, health care environment, and housing security). Publications highlighting the development and validation work supporting the other measures in the system are reported elsewhere [13-21].

## Methods

### Study Participants

We screened a total of 614 potentially eligible participants; of these, 292 were eligible and 225 were enrolled. The participants were enrolled from August 16, 2019, through March 5, 2020. Data for these analyses were collected as a part of a broader study designed to develop multiple PROs that capture important social determinants of health and behavior; data supporting this development work are reported elsewhere (ie, housing security [13], illness burden [14], medication adherence [15], and health care access [16]). Inclusion criteria for this study were broad and there were no specific exclusion criteria. To qualify, individuals had to have a diagnosis of T2DM, be at least 18 years of age, be fluent in English, and be able to provide informed consent. The participants completed surveys independently if they were able to correctly pronounce the first 10 words on the Wide Range Achievement Test Fourth Edition (WRAT4) reading subtest [22], and those with 1 or more errors were assisted with survey completion by a study coordinator. Potential participants were identified and recruited at Boston Medical Center (BMC), a safety-net health care system, through their clinical data warehouse (primary recruitment source), internal census reports from electronic health records (tertiary recruitment source), or local lists of individuals that had previously participated in research at BMC and had given permission to be contacted for future studies in T2DM (secondary recruitment source).

### Measures of Health-Seeking Behavior

The new health-seeking behavior PROs were developed using both qualitative and quantitative methodologies. The initial pool of items included content that examined the actions and inactions of persons with T2DM who perceive themselves as needing medical care. Briefly, this item pool was refined using feedback from patients with T2DM and professionals (in both T2DM and measurement development); items were written and revised using the Lexile framework to ensure they were no higher than a fifth-grade reading level, and a translatability review was completed to ensure that future measure translation into other languages would be possible. In this study, we tested the finalized item pool in a sample of individuals with T2DM. As a result, 4 new PROs were developed (the development process is detailed below). All of the PROs generate scores that are on a T-score metric (mean 50, SD 10); higher scores indicate more health-seeking behavior. FireStar Version 1.3.2 (SW Choi) [23] was used to generate computer adaptive test (CAT) scores when appropriate. Preliminary reliability and validity data are reported for all PRO CAT, short form (SF), and full measure scores.

### Data Collection

REDCap (Research Electronic Data Capture; Vanderbilt University), a HIPAA (Health Insurance Portability and Accountability Act)–compliant online data capture system, was used to collect survey response data. The WRAT4 reading subtest [22] was used to assess reading level. In all, 34.5% (212/614) of participants passed the WRAT4 and were able to complete study assessments independently; those with 1 or more errors on the first 10 words (13/614, 2.1%) completed the assessments with the assistance of a study coordinator. Data were collected in accordance with local institutional review boards (IRBs), and the participants were required to provide informed consent before study participation.

### Ethical Considerations

Approval was obtained from the ethics committee of BMC, which served as the single IRB of record for this study (H-38545). The University of Michigan IRBMED ceded to the BMC/BUMC IRB (HUM00165735). The procedures used in this study adhere to the tenets of the Declaration of Helsinki. We followed the activities described in the Agency for Healthcare Research and Quality Informed Consent and Authorization Toolkit for Minimal Risk Research. This toolkit was developed to facilitate the process of obtaining informed consent and HIPAA authorization from potential research participants. Specifically, for those patients who met all criteria for participation, the consent process included a discussion of (1) the purpose of the study; (2) IRB safeguards; (3) informed consent; (4) permission for phone contacts; (5) permission for medical record review; and (6) the use of their health information. A waiver of documentation of informed consent was approved for this study, as the research presented no more than minimal risk of harm to participants. To protect privacy, the information obtained from the participants was the minimum necessary to conduct the study. All study documents were identified with a unique study ID to protect confidentiality. The study ID was linked to a master-code list, which contained all direct identifiers and was stored on a password-protected, encrypted computer on the BMC secure network with access limited to BMC study staff. Consent forms were stored separately in a locked file cabinet. The private health information (PHI) collected from the clinical data warehouse was also password protected and stored on an encrypted and HIPAA-compliant BMC-issued computer. Only the minimally necessary PHI was gathered, and all PHI shared with the University of Michigan was transmitted through the secure BMC server on the HIPAA-compliant Box, Inc. platform in accordance with their data-sharing agreement. Finally, the participants received a total of US $75 compensation for their participation.

### Sample Size Requirements

There is evidence to indicate that a constrained graded response model (GRM) model is appropriate for sample sizes that are smaller than 500 [24]. Recommendations indicate that stable parameter estimates can be achieved with this model, given a minimum size of 200 [24,25]. These recommendations also support iterative Wald 2-based differential item functioning (DIF) testing when there is a minimum sample size of ~100 participants per each investigated population subgroup [26].

### Statistical Analyses

#### Item Bank Development

**Figure 1:**
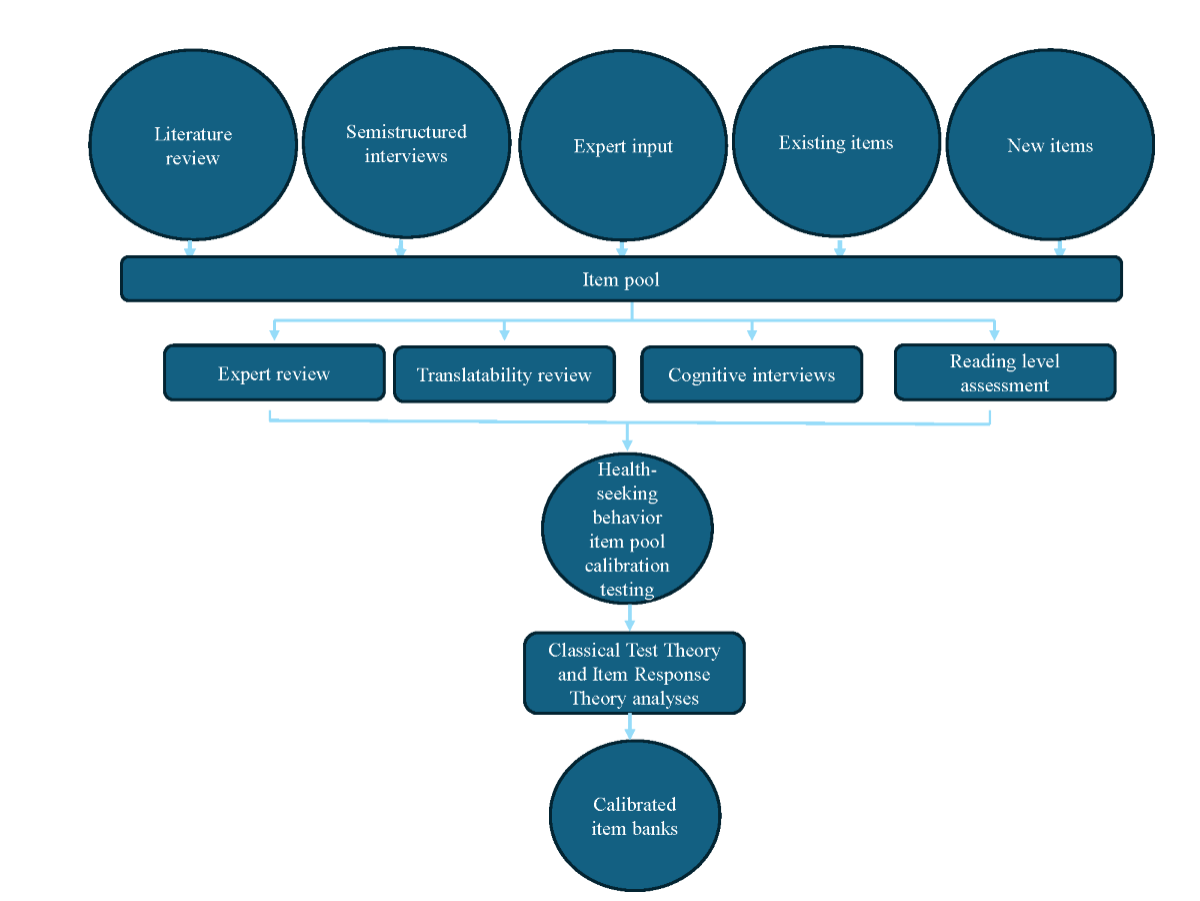
Process for new patient-reported outcome measurements development.

Following established measurement development standards [12], Classical Test Theory and Item Response Theory (IRT)–based analyses were used to inform the development of these new PROs (Figure 1). The initial item pool development is described elsewhere [27,28]. Specifically, general medical patients (n=37), caregivers (n=2), and care providers (n=9) completed semistructured interviews to identify concepts related to readmission risk. The item development process was iterative and included input from providers with expertise in T2DM and PRO measurement development; cognitive interviews with individuals with T2DM; reading level assessment (to ensure items were at fifth-grade reading level or below); and translatability review (ie, to facilitate future Spanish-language translation).

After the item pools were developed and administered to the 225 (36.6%) out of 614 participants, exploratory factor analysis (EFA; principal-axis factoring with a geomin [oblique] rotation) and confirmatory factor analysis (CFA; items were considered as categorical, interitem polychoric correlations were obtained and analyzed, a weighted least squares mean and variance adjusted estimator was used, and a pairwise-inclusion strategy was used for forming polychoric correlation matrices, which allowed for the incorporation of study participants with missing response data) were used to identify unidimensional sets of items (using Mplus version 7.4; LK Muthén and BO Muthén [29]) [30-32]. Essential unidimensionality would be supported by EFA if (1) the ratio of eigenvalue 1 to eigenvalue 2 is ≥4 and (2) ≥40% of item response variance is accounted for by eigenvalue 1. Items with (1) n<5 responses in any response category, (2) low item-adjusted total score correlations (ie, <0.40), or (3) nonmonotonicity (according to Testgraf Software; J Ramsay) [33] were candidates for exclusion. Essential unidimensionality would be supported by CFA: (1) comparative fit index ≥0.90, (2) Tucker-Lewis index ≥0.90, and (3) root mean square error of approximation (RMSEA) <0.15 [34-37]. Items with low factor loadings (lx <0.50), high residual correlations (>0.20), or high correlated error modification indices (≥100) were candidates for CFA-based exclusion [30-32].

Next, a constrained GRM [38], that is, a common-slope GRM, was estimated and used to obtain item parameters and identify item misfit (ie, S-X2 divided by *df* effect size >3 using IRTPRO) [39,40]. We also examined items for DIF: (1) candidates for exclusion exhibited statistically significant (*P*<.01) item parameter differences and (2) >2% of DIF-corrected versus uncorrected score differences were more than the uncorrected score SE (analyses were conducted in IRTPRO version 3.1.2 [L Cai, D Thissen, and SHC du Toit] [31] using iterative Wald-2 testing [41,42]). DIF was examined for four factors: (1) age (<60 vs ≥60 years), (2) sex (male vs female), (3) education (≤high school vs >high school), and (4) socioeconomic status (“have enough income to pay rent or mortgage” and “can afford to pay bills on time,” both categorized as never, rarely, sometimes, usually, and always). After the GRM and DIF analyses were completed and item parameters estimated, CFA was again used to confirm unidimensionality (according to the criteria presented above). As previously noted, the GRM analyses produced calibrated item parameters, that is, the slope and threshold estimates characteristic of an IRT-developed item bank. This item parameter information was also used to program CAT administration for measures or banks that included more than 10 items. CAT scores were simulated using FireStar software [23]; they were then used for analyses that examined preliminary reliability and validity. To begin, CAT item responses were simulated from a sample of 2000 cases drawn from a clinical population (ie, having a mean 50, SD 10 score in the direction of worse health status; ie, T-scores >60 for negatively worded concepts or T-scores <40 for positively worded concepts) so as to obtain a rigorous CAT performance assessment based on patients in a clinical context. CAT administration parameters (eg, number of items to administer and targeted score reliability level) were optimized to balance response burden and score precision. For calibrated measures or item banks that included greater than 6 items, 6-item SFs were created by balancing clinician input on item content with psychometric considerations, which included score-level reliability.

#### Preliminary Descriptive Data for the New PROs

PRO scores were normally distributed and supported the use of parametric data analyses. With regard to reliability, we estimated Cronbach α internal consistency for full-length measures and SFs (a priori criterion: α≥.70) [43]. The a priori criterion for acceptable floor and ceiling effects was ≤20% [44,45].

## Results

### Study Participants

A total of 225 participants with T2DM were included in this sample. Table 1 lists the sample descriptive data.

**Table 1:** Descriptive data for the study participants (N=225).

Variables		Values
**Age (years), mean (SD)**		57.7 (11)
**Sex, n (%)**		
	Female	118 (52)
	Male	107 (48)
**Ethnicity, n (%)**		
	Not Hispanic or Latino	187 (83)
	Hispanic or Latino	38 (17)
**Race, n (%)**		
	White	40 (18)
	Black or African American	169 (75)
	Other	16 (7)
**Education, n (%)**		
	Less than high school	42 (19)
	High school graduate or equivalent	73 (32)
	Some college, no degree	49 (22)
	Associate or vocational degree	29 (13)
	4-Year college degree	19 (8)
	Master’s degree or above	13 (5)
**Marital status, n (%)**		
	Single	123 (55)
	Married or cohabitating	34 (15)
	Separated or divorced	48 (21)
	Widowed	19 (8)
	Missing	1 (<1)
**Insurance Coverage,** **n (%)**		
	Medicare or Medicaid	178 (79)
	Commercial	40 (18)
	Other	7 (3)
	Charlson Comorbidity Index	4.4 (2.73)
**At the end of the month...,** **n (%)**		
	I do not have enough money to make ends meet	139 (62)
	I have enough money to make ends meet	66 (29)
	I have money left over	20 (9)
**Do you usually ask someone to help you read materials you receive from the hospital doctor?** **n (%)**		
	Yes	54 (24)
	No	169 (75)
	Missing	2 (1)
**HbA_1c_^a^(%), mean (SD)**		8.1 (2.2)

^a^HbA_1c_: hemoglobin A_1c_.

### Item Bank Development

The initial item pool contained 56 items. Table 2 includes the results of the EFA supporting a 4-factor model. The first factor included 6 items that generally represented contacting a primary care physician (PCP) for specific symptoms; the second factor included 13 items about general beliefs concerning health care and when it is appropriate or advisable to seek help from a PCP; the third factor included 5 items that generally represented contacting a friend or family member for health advice; and the fourth factor included 4 items that generally represented using the internet for researching and obtaining health advice. Table S1 in Multimedia Appendix 1 provides a summary of the iterative process that was used to identify these 4 different unidimensional item sets. A constrained (common slope) GRM did not identify any items with significant misfits in these 4-item sets. In addition, there were no items identified across item sets with impactful DIF. The final CFA (Table 3) supported the unidimensionality of these 4 separate PROs.

**Figure 2:**
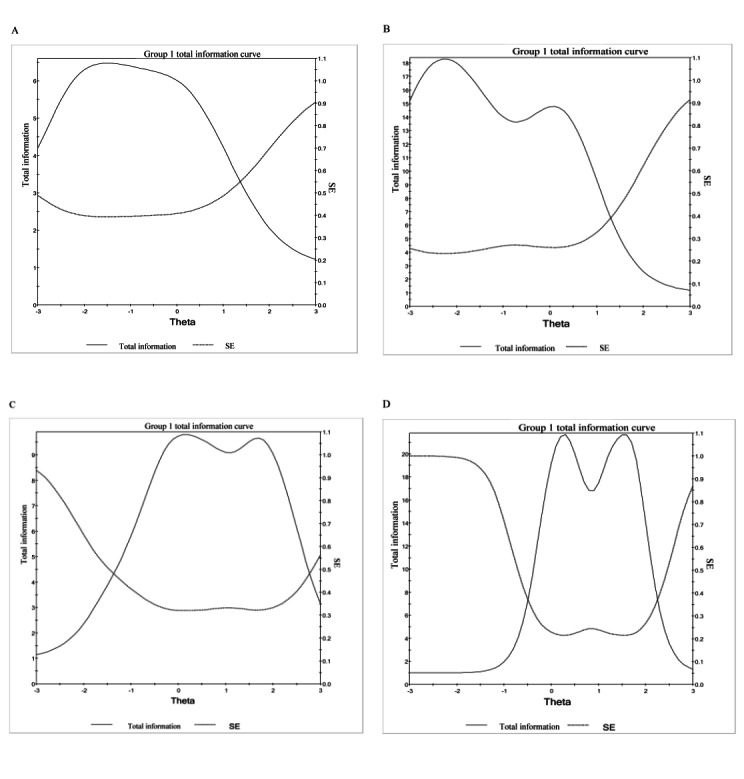
Health-seeking behavior measure test information plots: (A) Health-Seeking Behavior: PCP-Specific; (B) Health-Seeking Behavior: General Beliefs; (C) Health-Seeking Behavior: Family or Friends-Specific; and (D) Health-Seeking Behavior: Internet-Specific.

**Figure 3:**
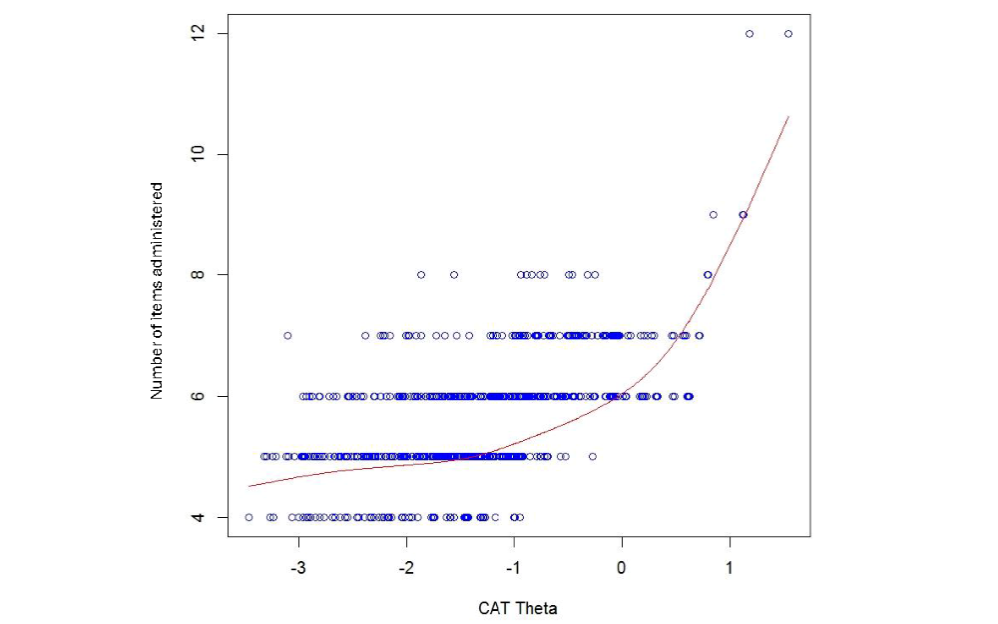
Health-Seeking Behavior: General Beliefs—number of CAT items by CAT theta. CAT: computer adaptive test.

The 4 new PROs included (1) Health-Seeking Behavior: PCP-Specific; (2) Health-Seeking Behavior: General Beliefs; (3) Health-Seeking Behavior: Family or Friends-specific; and (4) Health-Seeking Behavior: Internet-Specific. Item information is highlighted in Figure 2 for each of these PROs. A total of 3 of the 4 newly developed PROs are calibrated measures (Table 4), while Health-Seeking Behavior: General Beliefs is an item bank, which can be administered as a CAT or a 6-item SF (the items selected for inclusion in the Health-Seeking Behavior: General Beliefs SF are italicized in Table 4). With a minimum number of items as 4, a maximum number of items as 12, and a targeted score-level reliability of 0.85, the CAT tended to administer the minimum number of items from –3.5 SD units to –1.0 SD units. Conversely, the CAT administered the maximum number of items at ≥+1.2 SD units. Figure 3 illustrates the minimum and maximum number of items administered by the Health-Seeking Behavior: General Beliefs CAT. Tables S2-S5 in Multimedia Appendix 1 can be used to convert SF raw summed scores to T-scores, with their associated SEs.

**Table 2:** Exploratory factor analysis results for REDD-CATa Health-Seeking Behavior items. Italicized values indicate primary factor loadings.

REDD-CAT Health-Seeking Behavior item	Factor 1: PCP^b^ for health advice	Factor 2: General belief about when to seek out health advice	Factor 3: Family or friends for health advice	Factor 4: Internet for health advice
When I experience minor symptoms, I go to my PCP^c^	*0.613*	–0.049	0.004	0.004
When I experience serious symptoms, I contact my PCP^c^	*0.572*	0.164	0.011	–0.033
When I have questions about my medication(s), I ask my PCP^c^	*0.429*	0.416	0.023	–0.054
When I have health questions, I immediately call my doctor ^c^	*0.774*	–0.005	0.034	-0.143
When I call my PCP, I have specific questions^c^	*0.710*	0.102	–0.012	0.022
When I experience worrisome symptoms, I go to my PCP^c^	*0.589*	0.158	–0.050	0.126
If I have minor symptoms for more than a week I call my PCP^d^	0.379	*0.398*	0.034	–0.061
I feel confident asking questions about my health^d^	–0.144	*0.762*	–0.104	0.013
I trust the information I receive from my PCP about my health^d^	0.036	*0.753*	–0.027	–0.011
I call my PCP for advice about my health^d^	0.228	*0.677*	–0.026	–0.019
I set up an appointment with my PCP when I have questions about my health^d^	0.295	*0.571*	–0.071	0.075
My health is a top priority^d^	–0.012	*0.806*	0.020	–0.114
I make sure to ask my PCP questions when I don’t understand something^d^	–0.021	*0.885*	–0.031	–0.022
I reach out to my PCP when I have questions about my health^d^	0.215	*0.758*	0.051	0.006
I like staying informed about my health^d^	–0.084	*0.863*	–0.027	0.166
I seek the advice of my doctor to inform me about my health^d^	0.094	*0.859*	0.055	–0.045
I seek out ways to better my health^d^	0.002	*0.744*	0.054	0.104
I have someone to contact when I have questions about my health^d^	0.082	*0.531*	0.187	–0.056
I am confident about my abilities to answer questions about my health^d^	0.067	*0.671*	0.002	0.149
When I experience serious symptoms, I ask a friend or family member for advice^c^	–0.011	–0.008	*0.706*	0.000
When I have questions about my health, I ask my friends or family members to explain things^c^	–0.018	0.000	*0.836*	0.024
When I have questions about my medication(s), I ask friends or family members for assistance^c^	0.052	–0.006	*0.790*	0.084
When I experience worrisome symptoms, I ask my friends and family for advice^c^	0.041	–0.113	*0.849*	0.215
I reach out to friends and family members when I have questions about my health^d^	–0.173	0.130	*0.830*	–0.070
When I experience serious symptoms, I use the internet to find information^c^	–0.012	–0.038	0.049	*0.899*
When I experience minor symptoms, I use the internet to find information^c^	0.157	–0.127	–0.018	*0.933*
When I have questions about my health, I use the internet to find information^c^	–0.062	0.090	0.001	*0.958*
When I have questions about my medication(s), I use the internet for help^c^	–0.042	0.070	0.208	*0.767*

^a^REDD-CAT: Re-Engineered Discharge for Diabetes-Computer Adaptive Test.

^b^PCP: primary care physician.

^c^The response set for these items was never, rarely, sometimes, usually, and always.

^d^The response set for these items was strongly disagree, disagree, neither agree nor disagree, agree, and strongly agree.

**Table 3:** Final overall model fit and reliability characteristics for the Re-Engineered Discharge for Diabetes-Computer Adaptive Test (REDD-CAT) Health-Seeking Behavior measures.

Health-Seeking Behavior measure	Items, n	CFI^a^ (criterion ≥.90)	TLI^b^ (criterion ≥.90	CFA^c^-based RMSEA^d^ (criterion <.15)	SRMR^e^ (criterion <.08)	α reliability (criterion ≥.80)	IRT^f^-based RMSEA (criterion <.15)	Response pattern or person-centered reliability (criterion ≥.80)
PCP^g^-Specific	6	.994	.990	.048	.036	.780	.07	.80
General Beliefs	13	.962	.955	.099	.059	.908	.05	.90
Family or Friends-specific	5	.997	.995	.061	.027	.865	.06	.86
Internet-Specific	4	.998	.995	.120	.017	.908	.12	.78

^a^CFI: comparative fit index.

^b^TLI: Tucker-Lewis Index.

^c^CFA: confirmatory factor analysis.

^d^RMSEA: root mean square error of approximation.

^e^SRMR: standardized root mean residual.

^f^IRT: Item Response Theory.

^g^PCP: primary care physician.

In general, if the total information per score level is ≥3.3 and the resultant SE is ≤0.55, this will provide an acceptable score-level reliability of ≥0.70. These figures show acceptable total information and SE: (1) Health-Seeking Behavior: PCP-Specific theta scores between approximately –3.0 and 1.3 (T-scores between approximately 20 and 63); (2) Health-Seeking Behavior: General Beliefs theta scores between approximately –3.0 and 1.8 (T-scores between approximately 20 and 68); (3) Health-Seeking Behavior: Family or Friends-Specific theta scores between approximately –1.6 and 2.9 (T-scores between approximately 34 and 79); and (4) Health-Seeking Behavior: Internet-Specific theta scores between approximately –0.7 and 2.5 (T-scores between approximately 43 and 75).

Figure 3 shows the number of CAT items used for different score levels in SD units: from approximately –3.5 SD units to –1.0 SD units, the CAT tended to use the minimum of 4 items from the item bank; at approximately ≥+1.2 SD units, the maximum of 12 items from the item bank were used by the CAT.

**Table 4:** Item parameters for the Re-Engineered Discharge for Diabetes-Computer Adaptive Test (REDD-CAT) Health-Seeking Behavior: General Beliefs item bank. Items in italics were selected for the Health-Seeking Behavior 6-item Short Form.

Item	Slope^a^	Threshold 1^b^	Threshold 2^b^	Threshold 3^b^	Threshold 4
*If I have minor symptoms for more than a week, I call my PCP^c^*	2.16	–2.13	–1.47	–1.08	0.68
I feel confident asking questions about my health	2.16	–2.42	–2.32	–1.76	–0.18
*I trust the information I receive from my PCP about my health*	2.16	–3.01	–2.78	–1.85	–0.10
I call my PCP for advice about my health	2.16	–2.63	–1.67	–1.20	0.58
*I set up an appointment with my PCP when I have questions about my health*	2.16	–2.45	–1.54	–1.06	0.59
My health is a top priority	2.16	–3.35	–2.99	–2.58	–0.53
I make sure to ask my PCP questions when I don’t understand something	2.16	–3.10	–2.66	–2.37	–0.17
I reach out to my PCP when I have questions about my health	2.16	–3.04	–2.16	–1.83	0.28
I like staying informed about my health	2.16	–3.33	–2.75	–2.24	–0.10
I seek the advice of my doctor to inform me about my health	2.16	–3.02	–1.97	0.15	—
*I seek out ways to better my health*	2.16	–2.89	–2.26	–1.58	0.33
*I have someone to contact when I have questions about my health*	2.16	–2.15	–1.40	–0.99	0.80
*I am confident about my abilities to answer questions about my health*	2.16	–2.58	–1.96	–1.63	0.39

^a^Slopes are the discrimination parameters [which are held constant in a constrained (common slope) graded response model].

^b^Thresholds are the location (or difficulty parameters); they indicate the locations on the measurement continuum where an item can provide its most precise measurement. Thus, items with lower-value thresholds measure most precisely at those lower score values; such an item can therefore be thought of as an “easier” item. Items with higher threshold values measure most precisely at those higher score values; such an item can therefore be thought of as a “harder” item. All items use the following Likert response set: 1=strongly disagree, 2=disagree, 3=neither agree nor disagree, 4=agree, and 5=strongly agree.

^c^PCP: primary care physician.

### Preliminary Descriptive Data for the New PROs

Internal consistency reliability ranged from acceptable (≥0.70 for Health-Seeking Behavior: PCP-Specific and Health-Seeking Behavior: General Beliefs-SF) to good (≥0.80 for Health-Seeking Behavior: Family or Friends-Specific) to excellent (≥0.90 for Health-Seeking Behavior: General Beliefs-Full Bank, Health-Seeking Behavior: General Beliefs-CAT, and Health-Seeking Behavior: Internet-Specific). There were no significant floor or ceiling effects (Table 5).

**Table 5:** Descriptive data for the different Re-Engineered Discharge for Diabetes-Computer Adaptive Test (REDD-CAT) Health-Seeking Behavior patient-reported outcomes.

Health-Seeking Behavior	Patients, n	Internal consistency reliability	Score, mean (SD)	% at floor	% at ceiling
General Beliefs–CAT^a^	225	0.91	50 (9.1)	0	0
General Beliefs–SF^b^	225	0.78	49.9 (8.4)	0.4	7.6
General Beliefs Full Bank	225	0.91	50.1 (9.4)	0	0
PCP^c^-Specific Full Bank	225	0.78	50 (8.9)	0.4	8.4
Family or Friends-Specific Full Bank	225	0.87	50 (9.3)	8.9	0
Internet-Specific Full Bank	225	0.91	50 (9.1)	5.3	0

^a^CAT: Computer Adaptive Test.

^b^SF: Short Form.

^c^PCP: primary care physician.

## Discussion

### Principal Findings

The purpose of this study was to develop new PROs that capture patterns of health-seeking behaviors in persons with T2DM. Health-seeking behaviors may influence diabetes-related self-management decisions among patients that may lead to acute complications, poor health outcomes, and unplanned hospitalizations. As such, health-seeking behaviors should be explored as a potential social determinant of health and risk factor for hospital readmission.

Our findings support the development of four new measures banks: (1) Health-Seeking Behavior: PCP-Specific (6 items); (2) Health-Seeking Behavior: General Beliefs (13 items); (3) Health-Seeking Behavior: Family or Friends-Specific (5 items); and (4) Health-Seeking Behavior: Internet-Specific (4 items). These measures were developed for inclusion in the REDD-CAT measurement system, designed to assess important social determinants of behavior that are related to readmission risk in persons with T2DM. The REDD-CAT includes the first CATs developed specifically for use in T2DM; this includes the REDD-CAT Health-Seeking Behavior: General Beliefs-CAT. This CAT performs well, that is, 4-9 items administered, with reliability ≥0.85, for individuals with General Beliefs T-scores from 15 to 61. CATs administer more items to individuals with “extreme scores” (ie, scores at one or both ends of a measure’s scoring continuum; for General Beliefs, that would be T-scores ≥62). Thus, persons with T2DM having General Beliefs T-scores ≥62 would consistently need to take the maximum 12 items administered by a CAT (assuming its maximum number of items=12) in order to terminate the CAT scoring session. However, little to no gain in score precision would be achieved for those persons by the administration of additional items beyond 9 (ie, items 10-12). We therefore recommend setting the CAT “maximum items to administer” criterion to 9 items to ensure an adequate balance between precision and test burden when using this measure in populations where individuals report unusually high health-seeking behavior.

The new REDD-CAT Health-Seeking Behavior PROs were developed according to established methodology [12]; these new measures are homogenous (ie, they are composed of unidimensional item sets); have acceptable excellent psychometric reliability; and do not include items with a bias for age, sex, education, or socioeconomic status. All measures are scored on a T-score metric (mean 50, SD 10), with scores ≥60 indicating high health-seeking behavior (ie, ≥1 SD above average health-seeking behavior for persons with T2DM) and scores ≤40 indicating low health-seeking behavior (ie, ≥1 SD below average health-seeking behavior for persons with T2DM). For these new measures, more health-seeking behavior reflects “appropriate” behavior (ie, seeking out information when one is experiencing significant symptoms), whereas low health-seeking behavior indicates that the individual is not seeking out treatment, even though treatment might be warranted.

### Limitations

This study has several limitations. First, the CAT data represented were based on simulations and, therefore, need to be replicated in a sample that is tested using the actual CAT engine. In addition, generalizability may be limited given that the sample only included patients from a safety-net health system. Future work in other T2DM samples is needed to fully understand both the utility and specific relationship to readmission risk, as well as the strengths and weaknesses of these measures, including their overall validity, responsivity, and sensitivity.

### Conclusions

These new Health-Seeking Behavior PRO measures provide exciting tools for assessing self-reported health-seeking behavior in persons with T2DM. Furthermore, the relationship of these new measures, in conjunction with the rest of the REDD-CAT measurement system—which includes several measures of other important social determinants of health and behavior, including new measures of Housing Security [13], Illness Burden [14], Medication Adherence [15], Health Care Access [16], as well as measures from the HEAL (Healing Encounters and Attitudes List) measurement system [17], Neuro-QoL (Quality of Life in Neurological Disorders) [18,19], and PROMIS (Patient-Reported Outcomes Measurements Information System) [20,21])—provides a complementary arsenal of tools that can aid in identifying individuals with unmet social needs and those who are at increased risk for hospital readmission. Together, these new measures and the larger REDD-CAT system are designed to provide researchers and clinicians with a comprehensive toolkit to assess important social determinants of health and behavior related to readmission risk in patients with T2DM.

In sum, the REDD-CAT Health-Seeking Behavior measures provide a brief, reliable, and valid assessment of patients’ health-seeking behaviors and represent a marker for healthy coping amid the demands of diabetes care. This new measure can be used to aid in hospital discharge planning as a screening tool to identify those individuals with T2DM who are experiencing difficulties with the demands of diabetes management and require tailored education and support before leaving the hospital setting. In addition, although this measure was developed specifically for use in persons with T2DM, it may also have clinical use in other medical populations.

## Appendix

Multimedia Appendix 1 Unidimensional modeling and analyses, and conversion tables.

## Abbreviations

BMC: Boston Medical Center
CAT: computer adaptive test
CFA: confirmatory factor analysis
DIF: differential item functioning
EFA: exploratory factor analysis
GRM: graded response model
HEAL: Healing Encounters and Attitudes List
HIPAA: Health Insurance Portability and Accountability Act
IRB: institutional review board
IRT: Item Response Theory
Neuro-QoL: Quality of Life in Neurological Disorders
PCP: primary care physician
PRO: patient-reported outcome
PROMIS: Patient-Reported Outcomes Measurements Information System
REDCap: Research Electronic Data Capture
REDD-CAT: Re-Engineered Discharge for Diabetes-Computer Adaptive Test
RMSEA: root mean square error of approximation
SF: short forms
T2DM: type 2 diabetes mellitus
WRAT4: Wide Range Achievement Test Fourth Edition
