# Supplementary material for: Four New Patient-Reported Outcome Measures Examining Health-Seeking Behavior in Persons With Type 2 Diabetes Mellitus (REDD-CAT): Instrument Development Study
Source: JMIR Diabetes. 2024 Nov 22;9:e63434. doi: 10.2196/63434 (PMC11624447; doi:10.2196/63434)
Supplement: Multimedia Appendix 1 [file diabetes_v9i1e63434_app1.docx]

**Table S1.** Unidimensional modeling and analyses for the Re-Engineered Discharge for Diabetes-Computer Adaptive Test Health-Seeking Behavior item pool (item tracking history).

|  | | Unidimensional modeling | | | | | Initial item performance | | | IRT^a^ modeling | |  |
| --- | --- | --- | --- | --- | --- | --- | --- | --- | --- | --- | --- | --- |
| Domain | Item pool | EFA^b^ E1 divided by E2 ratio (criterion ≥4) | Percent of variance for E1 (criterion ≥40) | 1-factor CFA^c^ loading (criterion <.50) | 1-factor CFA residual correlation (criterion >.20) | 1-factor CFA modification index (criterion ≥100) | Item-adjusted total score correlations (criterion <.40) | Sparse cells (criterion<10) | Problems with monotonicity | IRT item misfit | DIF^d^ | Final number of items in bank |
| Health-Seeking Behavior: PCP^e^-specific | 15 items | 3.9 | 41.4 | 1 item | 0 items | 4 items | 4 items | 0 items | 0 items | 0 items | 0 items | 6 items |
| Health-Seeking Behavior: General Behavior | 25 items | 7.0 | 48.6 | 0 items | 3 items | 5 items | 4 items | 0 items | 0 items | 0 items | 0 items | 13 items |
| Health-Seeking Behavior: Family or Friends-Specific | 7 items | 6.2 | 67.3 | 0 items | 0 items | 2 items | 0 items | 0 items | 0 items | 0 items | 0 items | 5 items |
| Health-Seeking Behavior: Internet-Specific | 9 items | 4.2 | 57.4 | 0 items | 1 item | 3 items | 1 item | 0 items | 0 items | 0 items | 0 items | 4 items |

^a^IRT: Item Response Theory.

^b^EFA: Exploratory Factor Analysis.

^c^CFA: Confirmatory Factor Analysis.

^d^DIF: differential item functioning

^e^PCP: primary care physician

**Table S2.** Re-Engineered Discharge for Diabetes-Computer Adaptive Test Health-Seeking Behavior-General Beliefs item bank **6-item short form conversion table.**

| Raw summed score | T score | SE |
| --- | --- | --- |
| 6 | 18.43 | 3.99 |
| 7 | 21.25 | 3.83 |
| 8 | 22.92 | 3.80 |
| 9 | 24.56 | 3.68 |
| 10 | 26.17 | 3.55 |
| 11 | 27.62 | 3.50 |
| 12 | 29.00 | 3.46 |
| 13 | 30.35 | 3.43 |
| 14 | 31.66 | 3.43 |
| 15 | 32.96 | 3.45 |
| 16 | 34.28 | 3.48 |
| 17 | 35.61 | 3.53 |
| 18 | 36.98 | 3.60 |
| 19 | 38.43 | 3.68 |
| 20 | 39.89 | 3.75 |
| 21 | 41.50 | 3.84 |
| 22 | 43.18 | 3.92 |
| 23 | 44.88 | 3.98 |
| 24 | 46.72 | 3.91 |
| 25 | 49.06 | 3.85 |
| 26 | 51.65 | 3.80 |
| 27 | 54.38 | 3.80 |
| 28 | 57.34 | 3.94 |
| 29 | 60.92 | 4.41 |
| 30 | 66.13 | 5.56 |

**Table S3.** Re-Engineered Discharge for Diabetes-Computer Adaptive Test Health-Seeking Behavior-Primary Care Physician–specific **conversion table.**

| Raw summed score | T-score | SE |
| --- | --- | --- |
| 6 | 19.28 | 4.68 |
| 7 | 22.34 | 4.58 |
| 8 | 24.98 | 4.39 |
| 9 | 26.81 | 4.33 |
| 10 | 28.64 | 4.22 |
| 11 | 30.28 | 4.16 |
| 12 | 31.85 | 4.11 |
| 13 | 33.35 | 4.08 |
| 14 | 34.81 | 4.07 |
| 15 | 36.25 | 4.06 |
| 16 | 37.67 | 4.07 |
| 17 | 39.08 | 4.08 |
| 18 | 40.51 | 4.10 |
| 19 | 41.95 | 4.11 |
| 20 | 43.42 | 4.13 |
| 21 | 44.92 | 4.16 |
| 22 | 46.48 | 4.19 |
| 23 | 48.11 | 4.23 |
| 24 | 49.83 | 4.29 |
| 25 | 51.66 | 4.37 |
| 26 | 53.65 | 4.50 |
| 27 | 55.77 | 4.62 |
| 28 | 58.28 | 4.89 |
| 29 | 61.02 | 5.07 |
| 30 | 65.87 | 6.00 |

**Table S4.** Re-Engineered Discharge for Diabetes-Computer Adaptive Test Health-Seeking Behavior-Family or Friends-Specific **conversion table.**

| Raw summed score | T-score | SE |
| --- | --- | --- |
| 5 | 33.35 | 5.58 |
| 6 | 38.95 | 4.33 |
| 7 | 42.22 | 3.94 |
| 8 | 44.57 | 3.71 |
| 9 | 46.75 | 3.51 |
| 10 | 48.66 | 3.41 |
| 11 | 50.48 | 3.34 |
| 12 | 52.21 | 3.31 |
| 13 | 53.91 | 3.31 |
| 14 | 55.60 | 3.33 |
| 15 | 57.27 | 3.34 |
| 16 | 58.95 | 3.36 |
| 17 | 60.57 | 3.38 |
| 18 | 62.20 | 3.38 |
| 19 | 63.86 | 3.39 |
| 20 | 65.46 | 3.39 |
| 21 | 67.20 | 3.43 |
| 22 | 68.87 | 3.46 |
| 23 | 70.92 | 3.68 |
| 24 | 72.71 | 3.72 |
| 25 | 76.26 | 4.44 |

**Table S5.** Re-Engineered Discharge for Diabetes-Computer Adaptive Test Health-Seeking Behavior-Internet Specific **conversion table.**

| Raw summed score | T- score | SE |
| --- | --- | --- |
| 4 | 39.92 | 5.88 |
| 5 | 46.95 | 2.99 |
| 6 | 48.82 | 2.82 |
| 7 | 50.75 | 2.39 |
| 8 | 52.24 | 2.32 |
| 9 | 53.67 | 2.28 |
| 10 | 55.14 | 2.34 |
| 11 | 56.59 | 2.35 |
| 12 | 58.25 | 2.37 |
| 13 | 59.99 | 2.39 |
| 14 | 61.51 | 2.39 |
| 15 | 63.04 | 2.31 |
| 16 | 64.47 | 2.28 |
| 17 | 65.92 | 2.30 |
| 18 | 67.55 | 2.48 |
| 19 | 69.27 | 2.62 |
| 20 | 73.38 | 4.04 |
